# ^68^Ga-labeled fluorinated benzamide derivatives for positron emission tomography imaging of melanoma

**DOI:** 10.1371/journal.pone.0317489

**Published:** 2025-02-28

**Authors:** Chaewon Lee, Boreum Song, Eunsu Kim, Yejin Seo, Dagyeong Hong, Jiyu Kim, Wookyeong Jeong, Seong-Young Kwon, Dong-Yeon Kim, Ayoung Pyo

**Affiliations:** 1 College of Pharmacy and Research Institute of Pharmaceutical Science, Gyeongsang National University, Jinju, Korea; 2 CNCure Biotech, Hwasun, Korea; 3 Department of Nuclear Medicine, Chonnam National University Medical School and Hwasun Hospital, Hwasun, Korea; Università degli Studi di Brescia: Universita degli Studi di Brescia, ITALY

## Abstract

Malignant melanoma tends to aggressively metastasize, resulting in it being a potentially lethal form of skin cancer with high mortality rates. The advanced stages of melanoma have a very poor prognosis because of the high tendency for metastasis, and there is therefore, a strong desire to develop efficient technology for the early detection of melanoma. The benzamide structure, which contains aromatic ring and amine group, exhibits a high affinity for melanin, making it a promising agent for targeting melanoma in diagnostic and therapeutic applications. In this study, we synthesized a fluorinated benzamide derivative and chelated it with the radioisotope ^68^Ga to detect melanoma on positron emission tomography (PET) imaging, and then evaluated its biological properties. We synthesized the new probe 2,2’,2,”2”’-(2-(4-(3-(2-((2-(5-fluoropicolinamido)ethyl)(methyl)amino)ethyl)thioureido)benzyl)-1,4,7,10-tetraazacyclododecane-1,4,7,10-tetrayl)tetraacetic acid (MI-0202C1) for melanoma imaging from 5-fluoropyridine-2-carboxylic acid and conjugated 1,4,7,10-tetraazacyclododecane-1,4,7,10-tetraacetic acid (DOTA) for chelation with ^68^Ga. The radiochemical yield and radiochemical purity of the ^68^Ga chelation complex were confirmed by radio-thin layer chromatography (radio TLC). *In vitro* cellular uptake of ^68^Ga-MI-0202C1 was verified in B16F10 cells (murine melanoma)*. In vivo* distribution and small animal PET studies were conducted on mice bearing B16F10 xenografts. The MI-0202C1 was chelated with ^68^Ga at 90°C for 10 min at pH 5, resulting in a radiochemical yield and radiochemical purity of over 95%. The cellular uptake of ^68^Ga-MI-0202C1 over 60 min was higher in a group treated with *L*-tyrosine (2 mM) than in a non-treated group, indicating selective uptake of melanin. ^68^Ga-MI-0202C1 successfully visualized B16F10 xenografts in microPET imaging performed at 30 and 60 min after intravenous injection, suggesting that ^68^Ga-MI-0202C1 has considerable potential as a diagnostic probe for melanoma.

## Introduction

Malignant melanoma is a deadly skin cancer with a highly aggressive tendency to metastasize, and the American Cancer Society reported that there were approximately 97,610 new cases and 7,990 deaths in the United States in 2023 [1]. Early diagnosis and precise staging are critical in the management of melanoma because stages 1 and 2 are surgically treatable. By contrast, advanced stages present a very poor prognosis because of their high tendency to metastasize [2,3]. Consequently, the development of efficient early diagnostic technologies is essential to improve survival rates.

Positron emission tomography (PET) offers improved image quality and higher sensitivity and resolution in comparison with single-photon emission computed tomography (SPECT). 2-Deoxy-2-[^18^F]fluoro-D-glucose ([^18^F]FDG) is the only PET imaging probe currently utilized for melanoma detection in clinical settings, but it is constrained by two important limitations. First, it exhibits relatively low performance in the identification of subcentimeter lesions and micrometastases [4], and second, because the molecular targets of [^18^F]FDG are glucose transporter and hexokinase [5], the focus of this tracer is on reflecting the metabolic activity of the tumors, which can reduce its reliability for detecting melanoma. Hence, there is a strong need for novel PET probes for detecting small-sized or early-stage metastatic melanoma.

A notable feature of melanoma is the widespread expression of melanin in the majority of tumor cells [3]. The benzamide structure, which has recently been actively investigated as a melanoma imaging probe, demonstrates an affinity with selectivity for the pigment melanin [6], a biopolymer produced by melanocytes [7,8]. Numerous benzamide derivatives labeled with radioisotopes have been developed and evaluated for imaging melanoma [7,9–15], and these compounds have demonstrated significant potential as effective agents for targeting melanoma in diagnostic applications. Among the benzamide derivatives currently reported, a picolinamide derivative structure showed particularly high tumor uptake [16,17]. We believe that after adequate structural modification of these derivatives’ backbone, they could be used for imaging or treating malignant melanoma by changing the radioisotope paired with it (diagnostic/therapeutic radionuclide).

^68^Ga (t_1/2_ =  68 min) can be readily acquired from a ^68^Ge/^68^Ga generator without the requirement for synthesis facility and an expensive cyclotron [18]. The most common way to label Ga is by applying a bifunctional chelator, such as 1,4,7,10-tetraazacyclododecane-1,4,7,10-tetraacetic acid (DOTA), which is a widely used macrocyclic chelator for radioactive metal complexation [19]. In this study, we successfully synthesized ^68^Ga-chelated 2,2’,2,”2”’-(2-(4-(3-(2-((2-(5-fluoropicolinamido)ethyl)(methyl)amino)ethyl)thioureido)benzyl)-1,4,7,10-tetraazacyclododecane-1,4,7,10-tetrayl)tetraacetic acid (MI-0202C1) and performed biological evaluations to confirm its detection efficiency for malignant melanoma.

## Materials and methods

### Synthesis of *tert*-butyl (2-((2-(5-fluoropicolinamido)ethyl)(methyl)amino)ethyl)carbamate (1)

A solution of 2,2-diamino-*N*-methyldiethylamine (0.09 g, 0.77 mmol) in tetrahydrofuran (THF) (10 mL) was cooled to 0°C using an ice bath. To this ice-cooled solution, di-*tert*-butyl dicarbonate (Boc_2_O) (0.11 g, 0.51 mmol) was added in a dropwise manner to protect one of the amine groups. After this addition, the flask was removed from the ice bath and the reaction mixture was stirred at room temperature for 120 min. The reactant was evaporated to remove the solvent, and was subsequently dissolved in dimethylformamide (DMF). Then, 5-fluoropyridine-2-carboxylic acid (0.07 g, 0.51 mmol), *N,N,N*′*,N*′-tetramethyl-*O*-(*N*-succinimidyl)uronium tetrafluoroborate (TSTU) (0.15 g, 0.51 mmol), and *N,N-*diisopropylethylamine (DIPEA; 0.13 mL, 0.77 mmol) were sequentially added and the mixture was stirred at 60°C for 18 h. The crude mixture was extracted with dichloromethane (DCM) and the organic layer was dried over MgSO_4_. After evaporation of the solvent, 121 mg (69.20%) of product was obtained, and this was additionally purified using medium-pressure liquid chromatography (MPLC) (ethyl acetate:hexane:methanol =  1:2:0.5). The analytical data for compound (1) are: ^1^H-nuclear magnetic resonance (NMR) spectrum (400 MHz, CDCl_3_): δ (ppm) 1.43 (s, 9H), 2.34 (s, 3H), 2.62 (m, 4H), 3.25 (d, *J* =  5.1 Hz, 2H), 3.56 (q, *J* =  5.8 Hz, 2H), 7.54 (m, 1H), 8.24 (q, *J* =  8.7, 4.6 Hz, 1H), 8.46 (s, 1H). ^13^C-NMR spectrum (101 MHz, CDCl_3_): δ (ppm) 28.42, 36.56, 41.80, 56.05, 79.20, 123.83 (*J* =  18.5 Hz), 136.84 (*J* =  25.4 Hz), 146.48 (*J* =  3.7 Hz), 156.07, 163.26. The low-resolution mass spectrometry (LRMS) (ESI) *m/z* calculated for C_16_H_25_FN_4_O_3_ [M + H]^+^= 341.2, found 341.2.

### Synthesis of 2,2’,2,”2”’-(2-(4-(3-(2-((2-(5-fluoropicolinamido)ethyl)(methyl)amino)ethyl)thioureido)benzyl)-1,4,7,10-tetraazacyclododecane-1,4,7,10-tetrayl)tetraacetic acid (MI-0202C1)

To proceed with the next reaction, the Boc group was removed using trifluoroacetic acid (TFA) and DCM (1/10, v/v), and the TFA was then removed using an evaporator. After dissolving it in dimethyl sulfoxide (DMSO), 1.60 mg was transferred to a reaction vial. Then, a DMSO solution containing (*p*-SCN-Bn)-DOTA (1 eq, 0.01 mmol) was added to the reaction vial and the pH was adjusted to basic with DIPEA. The reaction was performed at room temperature for 12 h. The product was purified by semi-preparative high-performance liquid chromatography (HPLC) (gradient 5% to 95% of acetonitrile containing 0.1% TFA for 30 min and 95% to 5% of H_2_O containing 0.1% TFA; flow rate of 3 mL/min). The analytical data for MI-0202C1 is: high-resolution mass spectrometry (HRMS) (FAB) *m/z* calculated for C_35_H_50_FN_9_O_9_S [M + H]^+^= 792.3436, found 792.3517.

### Preparation of reference compound and thermal stability test

Chelation of Ga with 2,2’,2’‘,2’“-(2-(4-(3-(2-((2-(5-fluoropicolinamido)ethyl)(methyl)amino)ethyl)thioureido)benzyl)-1,4,7,10-tetraazacyclododecane-1,4,7,10-tetrayl)tetraacetic acid was performed by modifying a previously reported method [12]. The precursor was dissolved in 1.0 M sodium acetate (NaOAc) buffer (pH 5), and GaCl_3_ was dissolved in deionized water. The solutions were then mixed in a molar ratio of 1:2 in a vial set at 90°C. After confirming a pH of 5, the mixture was stirred at 90°C–95°C for 30 min. The reaction mixture was separated by semi-preparative HPLC.

To assess the stability of the precursor and the reference compound in a known ^68^Ga radiolabeling protocol [12,19], we subjected them to heating at 90°C for 10 and 30 min, followed by measurement of peak changes using semi-preparative HPLC.

### Radiolabeling of MI-0202C1

[^68^Ga]GaCl_3_ was eluted from the ^68^Ge/^68^Ga generator using 0.6 M HCl. After evaporation, the pH was first adjusted using 1.0 M NaOAc buffer (pH 5). The precursor (100 μg) was dissolved in water in a reaction vial, 1.0 M NaOAc buffer (pH 5) was added, and the mixture was heated at 90°C for 10 min. The radiochemical yield and radiochemical purity were confirmed by radio-thin layer chromatography (radio TLC) using a 0.1 M aqueous solution of anhydrous sodium carbonate (Na_2_CO_3_).

### Measurement of partition coefficient and stability studies

The ^68^Ga-MI-0202C1 (7.4 MBq) was dissolved in a round-bottom flask containing 3 mL of n-octyl alcohol and 3 mL of saline. The mixture was stirred vigorously for 20 min at room temperature and subsequently transferred into polypropylene tubes. The tubes were centrifuged at 14,000 RPM for 5 min. 50 μL of each layer from the aqueous and the n-octyl alcohol layers were transferred and counted on a gamma counter. The log D value was determined from quadruplicate samples.

The ^68^Ga-MI-0202C1 (5.14 MBq, 32 μL) was incubated with human serum (1 mL) at 37°C for 120 min. The stability of the radiotracer was evaluated by radio TLC developed with 0.1 M aqueous solution of Na_2_CO_3_ at 60 and 120 min after incubation. All experiments were performed in quintuplicate. An aliquot of the aqueous solution containing cysteine and histidine (both at 10 equivalents relative to the precursor, with volumes of 7.0 μL and 5.7 μL, respectively) was added to a solution of ^68^Ga-MI-0202C1 (18.5 MBq, 14 μL) at room temperature. Deionized water was added to each mixture to bring the total volume to 60 μL. Radio TLC analyses of the solutions were performed for 30, 60, and 120 min using a 0.1 M aqueous solution of Na_2_CO_3_ as eluent.

For the *in vivo* stability test, ^68^Ga-MI-0202C1 (37 MBq) was injected into the tail vein of B16F10-bearing mice. At 30 and 60 min post-injection, the blood sample was centrifugated at 14,000 RPM for 5 min. The supernatant was separated and analyzed by radio TLC, with a 0.1 M Na_2_CO_3_ solution as the mobile phase.

### *In vitro* cellular uptake study

B16F10 (mouse melanoma) cells were cultured in Dulbecco’s modified Eagle medium with 10% fetal bovine serum and 1% antibiotic-antimycotic solution. B16F10 cells (5 ×  10^4^ per well) were plated onto a 24-well plate and pretreated with *L*-tyrosine (2 mM) for 24 h. Non-treated cells were used as a control. A 0.5 mL volume of ^68^Ga-MI-0202C1 (0.74 MBq) was added to each well. After incubation for 30 or 60 min at 37°C, the medium was removed from each well. The cells were then washed twice with DPBS and trypsinized. The radioactivity of the supernatant and trypsinized cells was measured using a gamma counter. The data were plotted as CPM (counts per minute) ±  SD, calculated by correcting the radioactivity in the cell pellet for the number of cells.

### MicroPET imaging and biodistribution studies

The animal experiments were carried out following the National Institute of Health Guidelines on the Use of Laboratory Animals (National Academies Press, 2011) and the protocol approved by the Gyeongsang National University Institutional Animal Care and Use Committee for animal research (GNU-231204-M0221-01). All animal experiments were performed by personnel certified in the Animal Care and Use program. The tumor size and body weight were monitored every two days. The tumor model was induced by subcutaneous injection of B16F10 cells (5 ×  10^5^) into the right shoulder. After 9 days, the B16F10 subcutaneous tumor models were used for imaging (tumor size: 100–150 mm^3^). Anesthesia was performed using 2% isoflurane to inject cells and microPET imaging. Two weeks after the completion of microPET imaging, mice with tumor sizes of 1,500–2,000 mm³ or a 20% loss in body weight were euthanized *via* CO_2_ inhalation, with all efforts made to minimize suffering. The small animal PET images were acquired using a high-resolution small animal PET-SPECT-CT scanner (Inveon, Siemens Healthineers). MicroPET studies of ^68^Ga-MI-0202C1 (7.4 MBq) were conducted at 30 and 60 min after intravenous (*i.v.*) injection of the tracer into B16F10-bearing mice, with coronal images being acquired for 10 min. Additional quantitative analysis was performed using regions of interest that were drawn over the heart, lung, liver, stomach, intestine, kidney, muscle, bone, brain, skin, and tumor on decay-corrected coronal images of ^68^Ga-MI-0202C1 uptake in the mice. *Ex-vivo* biodistribution studies were performed 60 min after injection of ^68^Ga-MI-0202C1 (7.4 MBq) intravenously into B16F10 tumor-bearing models. Tumor, blood, and the 12 organs (heart, lung, liver, spleen, stomach, intestine, kidney, pancreas, normal muscle, bone, brain, and skin) were weighed, and the radioactivity of the collected tissue was measured using a gamma counter. Data in microPET images and biodistribution are expressed as the percentage of the injected dose per gram (%ID/g, n >  3).

### Statistical analysis

Statistical analysis was performed using the Mann-Whitney *U* test for unpaired data. The 95% confidence level was chosen to determine the significance of the difference in cellular uptake between ^68^Ga-MI-0202C1 with and without 2 mM *L*-tyrosine at 30 and 60 min. Differences were deemed significant if they exceeded the 95% confidence level (*P* <  0.05). All data are expressed as mean ±  SD.

## Results

### Chemistry and radiochemistry

**Fig 1:**
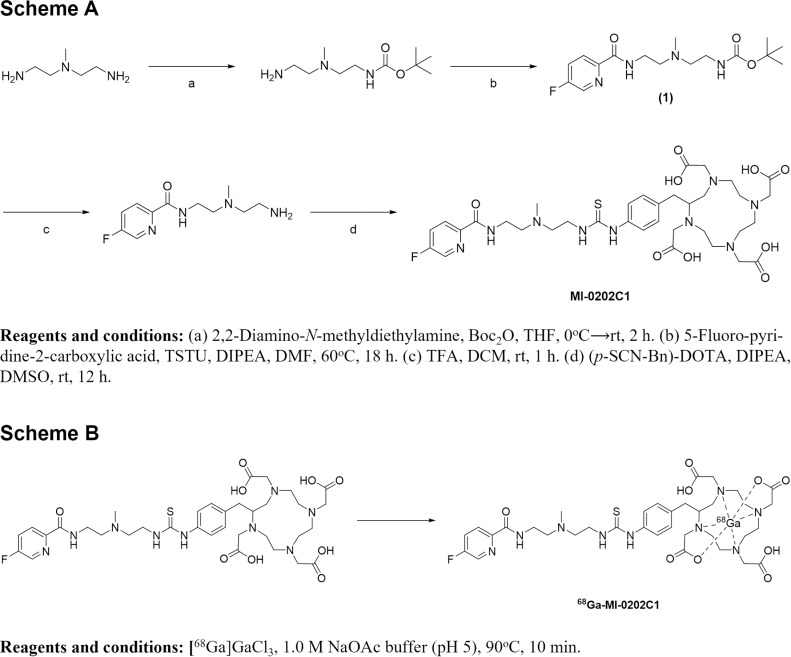
Synthesis of MI-0202C1 (Scheme A) and ^68^Ga-MI-0202C1 (Scheme B) (see text for details).

The synthesis scheme for MI-0202C1 is shown in Scheme A (Fig 1). First, compound (1) was synthesized *via* a reaction of 2,2-diamino-*N*-methyldiethylamine with 5-fluoropyridine-2-carboxylic acid, and was then separated by MPLC. The structural identity of compound (1) was analyzed using ^1^H, ^13^C NMR (S1 and S2 Figs) and low-resolution mass spectrometry. MI-0202C1 was prepared by conjugation with (*p*-SCN-Bn)-DOTA after removing the subsequent protecting group from compound (1). The precursor form was purified by semi-preparative HPLC (S3 Fig) and analyzed by HRMS to confirm its identity (C_35_H_50_FN_9_O_9_S [M + H]^ +^ =  792.3517). The final chemical yield of MI-0202C1 was 41.1 ±  13.3% (n =  5), with a purity exceeding 94% after semi-preparative HPLC. The reference compound was successfully produced by chelation with GaCl_3_ at 90°C–95°C for 30 min. The precursor and reference compounds were stable when heated at 90°C–95°C for 10–30 min.

^68^Ga-MI-0202C1 was formed by successfully chelating [^68^Ga]GaCl_3_ to MI-0202C1 at 90°C for 10 min, as shown in Scheme B (Fig 1). Subsequent radio TLC analysis confirmed that the tracer’s radiochemical yield and radiochemical purity both exceeded 95% (S4 Fig). The radiochemical purity of ^68^Ga-MI-0202C1 measured by the analytical HPLC system was reproduced to the results measured with radio TLC ( > 95%) (S5 Fig). The specific activity of the radiotracer was >  5.98 GBq/μmol. ^68^Ga-MI-0202C1 was used without any pH adjustment or further purification.

### Partition coefficient and stability studies

**Fig 2:**
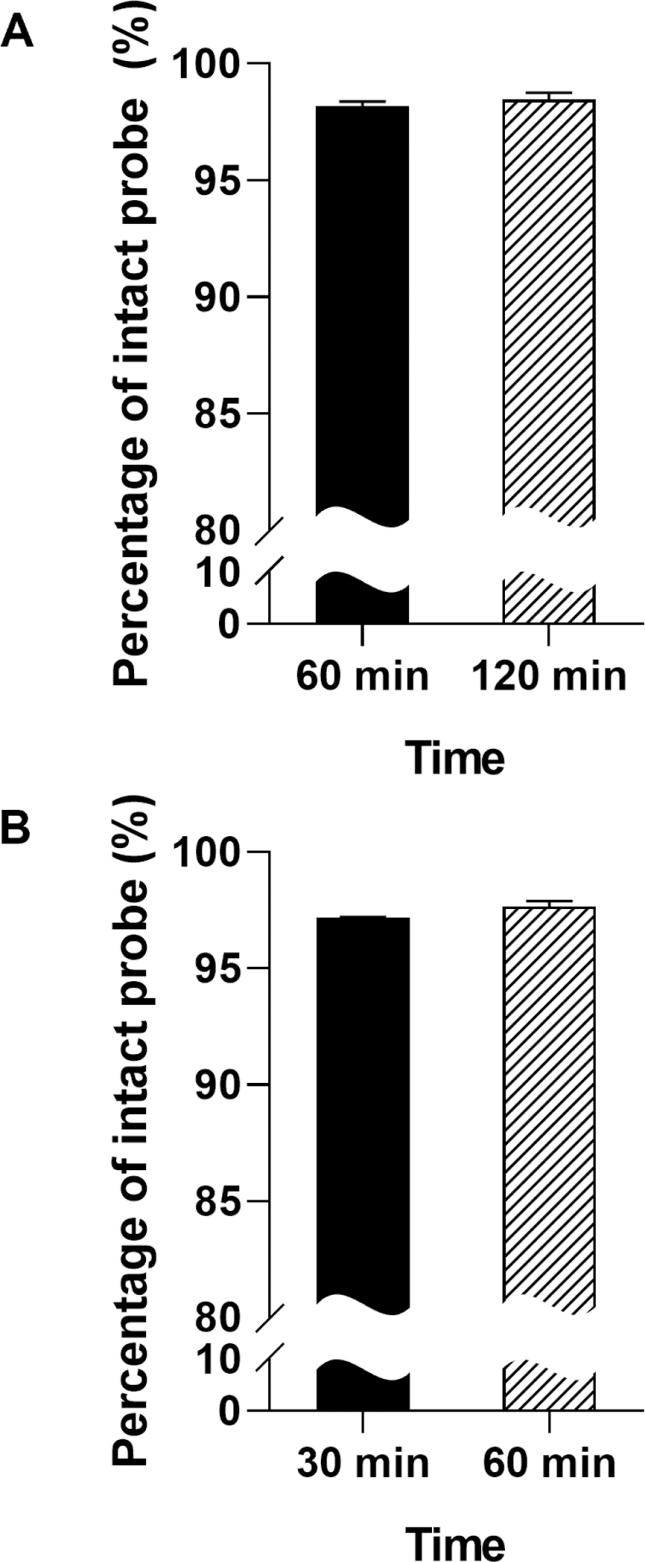
Stability of ^68^Ga-MI-0202C1. (A) *In vitro* stability in human serum (B) *In vivo* stability in mouse plasma.

The log D value of the ^68^Ga-MI-0202C1 was −3.82 ±  0.06, indicating that it is hydrophilic. The challenge experiments were performed to measure the stability of radiotracers in the solutions of histidine or cysteine [20]. Radio TLC did not show the release of free ^68^Ga from the ^68^Ga-MI-0202C1 at room temperature at 30, 60, and 120 min, respectively (S6 Fig). When the ^68^Ga-MI-0202C1 was incubated in human serum at 37°C for 120 min, the percentage of DOTA chelated form was greater than 95% (Fig 2A) (98.18 ±  0.20% at 60 min, 98.46 ±  0.30% at 120 min, respectively). No metabolite was detected in the serum of mice 60 min after *i.v.* injection (Fig 2B) (% of intact probe: 97.19 ±  0.02% at 30 min, 97.66 ±  0.24% at 60 min, respectively). Therefore, these results demonstrated sufficient stability of ^68^Ga-MI-0202C1 for the *in vivo* evaluation.

### *In vitro* cell uptake studies

**Fig 3:**
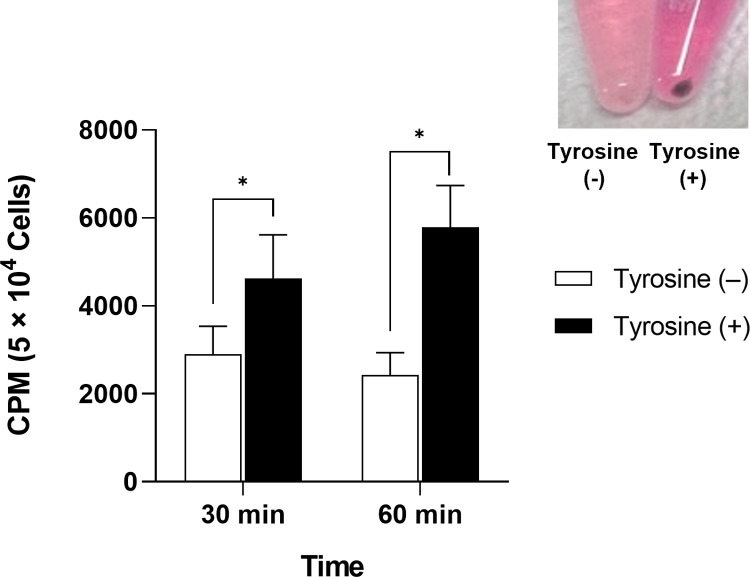
*In vitro* cellular uptake of ^68^Ga-MI-0202C1 in B16F10 melanoma cells. The image shows a B16F10 pellet in the absence (left) or presence (right) of *L*-tyrosine treatment after 24 h. *  indicates a *P* value <  0.05.

Cellular uptake studies of ^68^Ga-MI-0202C1 were conducted at 30 and 60 min after injection using the B16F10 cell line at 37°C (Fig 3). Treatment with *L*-tyrosine (2 mM) was used to activate melanoma cells and stimulate the melanogenesis pathway, and the B16F10 cells were observed turning black. The activated B16F10 cells exhibited higher accumulation of ^68^Ga-MI-0202C1 at both time points compared with the untreated controls (4,625.17 ±  992.00 CPM vs 2,901.76 ±  631.85 CPM, respectively, at 30 min; 5,787.23 ±  946.21 CPM vs 2,427.41 ±  507.40 CPM, respectively, at 60 min), with uptake increasing over time (*P* <  0.05).

### MicroPET imaging and biodistribution studies

**Fig 4:**
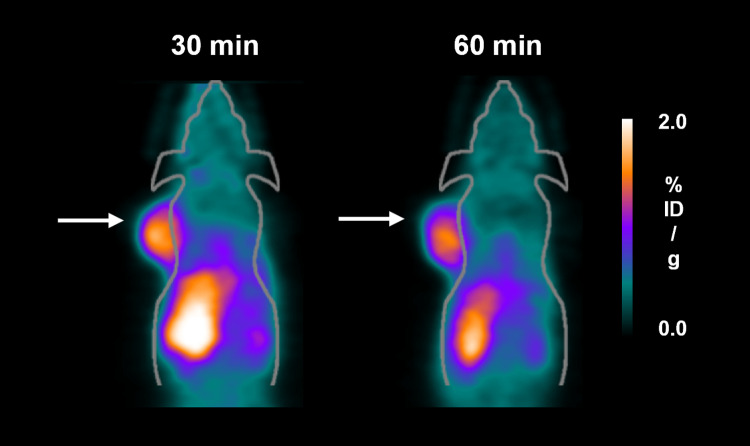
MicroPET images of ^68^Ga-MI-0202C1 uptake in a B16F10-bearing mouse at 30 and 60 min post-injection. The white arrow points to the tumor lesions.

A coronal microPET study was performed using B16F10 tumor-bearing mice, with images being obtained at 30 and 60 min after injection of the tracer *via* the tail vein. ^68^Ga-MI-0202C1 imaged B16F10 tumors with excellent tumor-background contrast at 30 min after injection, with this contrast remaining in the tumor until 60 min (tumor uptake: 1.12 ±  0.37%ID/g at 30 min, 1.02 ±  0.42%ID/g at 60 min, respectively) (Fig 4). High kidney uptake indicates this radiolabeled compound is primarily excreted through the kidney. Additionally, observation of the uptake of ^68^Ga-MI-0202C1 in the liver and intestine suggests that a portion of the compound undergoes hepatobiliary clearance. We also conducted additional quantitative analysis of ^68^Ga-MI-0202C1 in small animals (Table 1). The liver, intestine, and kidney showed similar uptake values at 30 and 60 min (liver uptake: 1.90 ±  0.54%ID/g at 30 min, 1.85 ±  0.57%ID/g at 60 min; intestine uptake: 2.52 ±  0.77%ID/g at 30 min, 2.54 ±  1.14%ID/g at 60 min; kidney uptake: 3.61 ±  0.79%ID/g at 30 min, 3.66 ±  1.73%ID/g at 60 min, respectively). Meanwhile, the uptake values in normal lung, bone, brain and skin consistently remained low. The tumor-to-lung, tumor-to-bone, tumor-to-brain and tumor-to-skin ratios at 60 min after ^68^Ga-MI-0202C1 injection were 1.34 ±  0.24, 3.66 ±  0.56, 3.92 ±  0.76 and 4.05 ±  1.64, respectively. *Ex-vivo* biodistribution studies were performed on the B16F10 tumor models at 60 min post-injection. The biodistribution results tended to be comparable to those observed in microPET images, except for intestinal uptake (S1 Table). The high intestinal uptake suggests rapid elimination of the tracer through the excretory system. Furthermore, low uptake in blood and normal organs such as lung, bone, brain, and skin suggest a favorable tumor-to-background ratio, a key feature of a promising diagnostic probe.

**Table 1:** Biodistribution studies of ^68^Ga-MI-0202C1 in B16F10 tumor-bearing mice at 30 and 60 min after *i.v.* injection.

	30 min	60 min
Heart	1.39 ± 0.11	1.30 ± 0.13
Lung	0.87 ± 0.29	0.76 ± 0.23
Liver	1.90 ± 0.54	1.85 ± 0.57
Stomach	0.78 ± 0.40	0.92 ± 0.54
Intestine	2.52 ± 0.77	2.54 ± 1.14
Kidney	3.61 ± 0.79	3.66 ± 1.73
Muscle	0.35 ± 0.14	0.34 ± 0.15
Bone	0.32 ± 0.14	0.27 ± 0.06
Brain	0.27 ± 0.07	0.26 ± 0.06
Skin	0.29 ± 0.06	0.26 ± 0.06
Tumor	1.12 ± 0.37	1.02 ± 0.42
	**60 min**	
Tumor-to-lung	1.34 ± 0.24	
Tumor-to-bone	3.66 ± 0.56	
Tumor-to-brain	3.92 ± 0.76	
Tumor-to-skin	4.05 ± 1.64	

## Discussion

Melanin is a biopolymer that contributes to homeostasis, photoregulation, thermoregulation, and defense against ultraviolet damage [4]. The presence of melanin in tumors complicates chemotherapy or radiotherapy because of its protective properties [21], and it is associated with disease advancement and poor survival. Most primary melanoma tumors contain melanin because of the increased melanogenesis caused by overexpression of tyrosinase in melanoma cells [22]. Interestingly, earlier studies found increased accumulation of radiolabeled benzamide derivatives in melanin-rich tissues [23]. Benzamide derivatives exhibit a selective affinity for melanin that is greatly increased in malignant melanoma. They are composed of three parts: a benzamide that can *π*-*π* interact with the heteroaromatic ring of melanin, an amine residue that can form an ionic interaction with the carboxylate of melanin, and an aliphatic liker that can connect the two components [16]. MI-0202C1 also has these parts within its chemical structure that are predicted to exhibit selective affinity for melanin, a specific malignant melanoma biomarker.

Previous research showed that tumor uptake of structures based on picolinamide derivatives [13,16,24] was higher than that of structures based on benzamide derivatives. In addition, the chemical reactivity of structures in which the nitrogen in the heteroaromatic ring is at position 3 is lower than structures where it is at position 2 or 4 [13]. As a result, *in vivo* stability is increased, resulting in improved tumor absorption and the maintenance of high tracer levels. *N*-(2-(dimethylamino)ethyl)-5-[^18^F]fluoropicolinamide ([^18^F]DMPY2) synthesized on the basis of these findings showed extremely high uptake in tumors (24.8%ID/g at 60 min) [16]. However, the ^18^F nuclide requires an expensive medical cyclotron. By contrast, ^68^Ga is a nuclide that can be easily obtained from ^68^Ge/^68^Ga generators, and it therefore costs less, and is especially attractive for facilities that do not have a cyclotron [19,25]. In this study, we assumed that the affinity for melanin comes from the fluoropyridine moiety, and that connection to a DOTA chelator would allow easy chelation with ^68^Ga by changing the amine residue moiety. Subsequently, we evaluated whether ^68^Ga-labeled MI-0202C1 could be used as a malignant melanoma targeting agent using the ^68^Ga/^177^Lu theranostic pair [26]. Furthermore, MI-0202C1 has shown stability under high-temperature conditions, making it an appropriate candidate for a ^68^Ga-chelated DOTA-based radiotracer.

^68^Ga-MI-0202C1 was synthesized relatively quickly (within 10 min) with high radiochemical yield and radiochemical purity. *In vivo* imaging demonstrated that ^68^Ga-MI-0202C1 showed strong potential as a PET tracer for detecting melanoma; *in vitro* results also showed more selective accumulation in activated B16F10 cells treated with *L*-tyrosine than in non-treated cells. ^68^Ga-SCN-DOTA-procainamide, a ^68^Ga-labeled benzamide derivative reported in 2012, showed similar tumor uptake values to ^68^Ga-MI-0202C1, but showed higher uptake values in some normal organs at 30 and 60 min. By comparison, ^68^Ga-MI-0202C1 showed generally low background uptake and significantly lower uptake in normal lung, brain, and bone than in tumor. Since the lung, bone, brain, and skin are the organs to which malignant melanoma commonly metastasizes [27], the low accumulation of ^68^Ga-MI-0202C1 in these organs presents a great advantage for its use as an agent to detect malignant melanoma.

^68^Ga-MI-0202C1 showed relatively lower uptake in B16F10 tumors than existing radiolabeled benzamide derivatives, despite being based on a structure with high tumor uptake. The reason for this may be the high hydrophilicity of the DOTA chelator [28]. Although hydrophilic radiopharmaceuticals are rapidly eliminated from non-target tissues, they may show lower tumor uptake than expected. Earlier studies have shown that structures with lower lipophilicity in the *N,N*-dialkylated benzamide series are associated with reduced melanoma uptake [29]. To enhance the drug’s lipophilic nature, alkyl chains [30,31] or benzene rings [32] could be added to the structure. Additionally, PEGylation, which can increase the blood circulation time [33,34], could be introduced to improve tumor uptake.

## Conclusion

The ^68^Ga-labeled fluorinated benzamide derivative called ^68^Ga-MI-0202C1 was successfully synthesized for use as a tracer for detecting melanoma using PET, and its biological properties were evaluated. The cellular uptake of ^68^Ga-MI-0202C1 was higher in *in vitro* melanoma cell culture with the presence of *L*-tyrosine than in untreated cells, indicating selective uptake by melanin. ^68^Ga-MI-0202C1 successfully visualized B16F10 tumors on microPET, and showed minimal accumulation in normal lung, bone, brain, and skin. These characteristics reveal its excellent potential for effective detection of malignant melanoma using PET.

## Supporting information

S1 Fig ^1^H NMR of compound (1). (TIF)

S2 Fig ^13^C NMR of compound (1). (TIF)

S3 Fig HPLC chromatogram of MI-0202C1. (TIF)

S4 Fig Radio TLC using 0.1 M Na_2_CO_3_ aqueous solution as the eluent. (a) Free ^68^Ga (b) ^68^Ga-MI-0202C1.
(TIF)

S5 Fig Radio HPLC chromatogram of ^68^Ga-MI-0202C1. (TIF)

S6 Fig Radio TLC of the stability of ^68^Ga-MI-0202C1 in excess L-cysteine (A) and L-histidine (B) solutions. (Arrow: ^68^Ga-MI-0202C1).
(TIF)

S1 Table *Ex-vivo* biodistribution studies of ^68^Ga-MI-0202C1 in B16F10 tumor-bearing mice at 60 min post-injection. (DOCX)
