# Supplementary figures and images for: 68Ga-labeled fluorinated benzamide derivatives for positron emission tomography imaging of melanoma
Source: PLoS One. 2025 Feb 28;20(2):e0317489. doi: 10.1371/journal.pone.0317489 (PMC11870364; doi:10.1371/journal.pone.0317489)

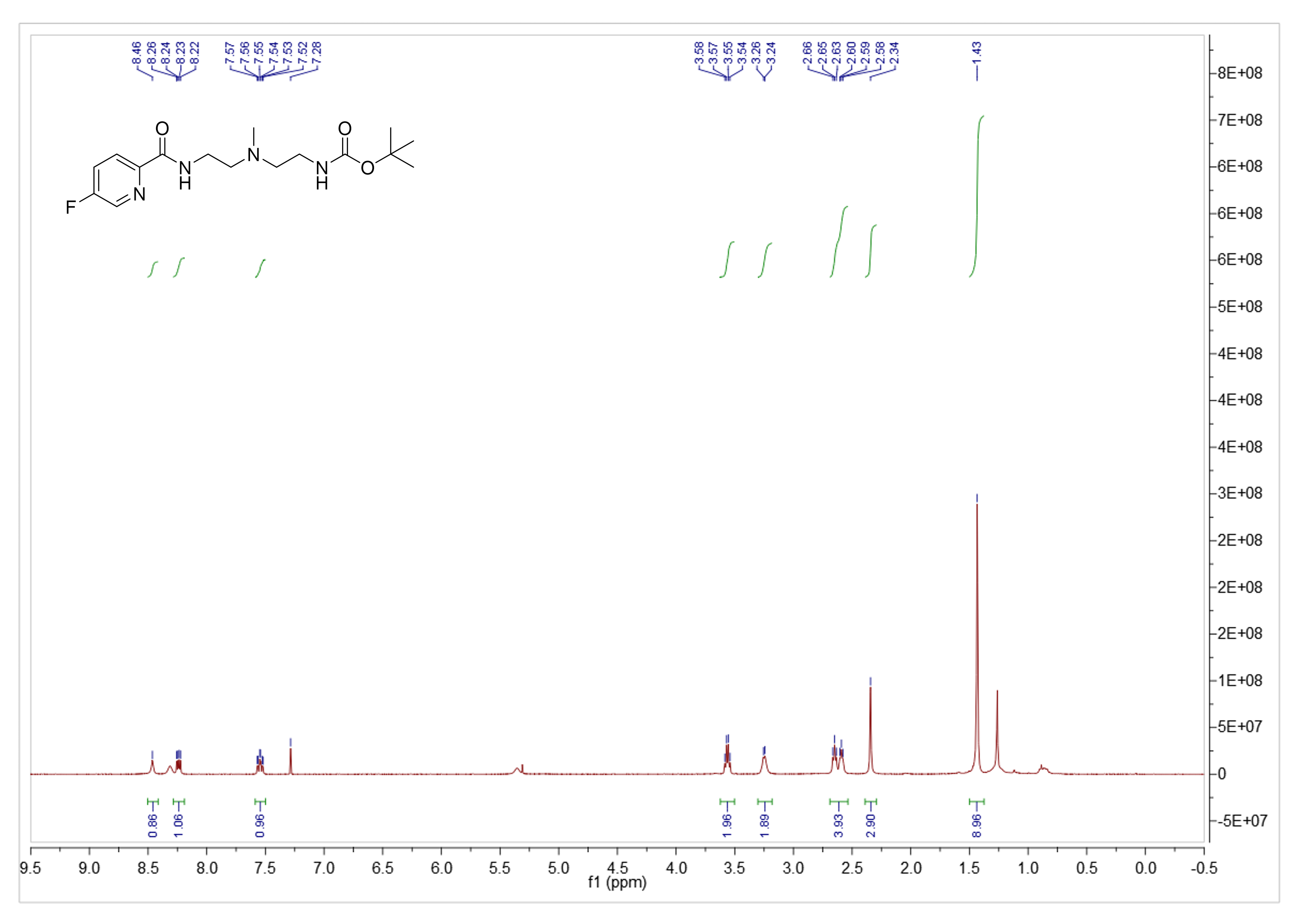

Supplement: S1 Fig — (TIF) [file pone.0317489.s001.tif]

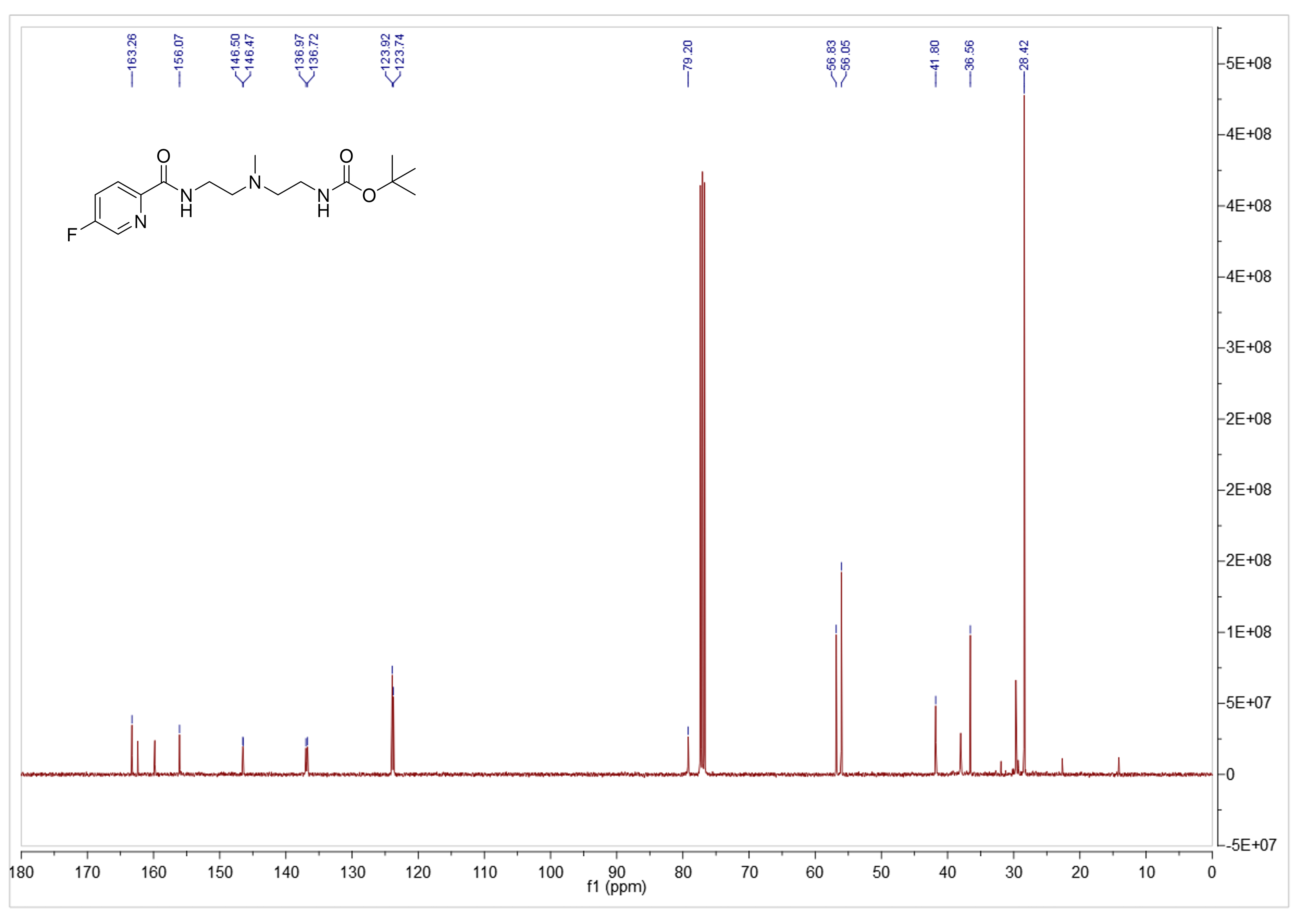

Supplement: S2 Fig — (TIF) [file pone.0317489.s002.tif]

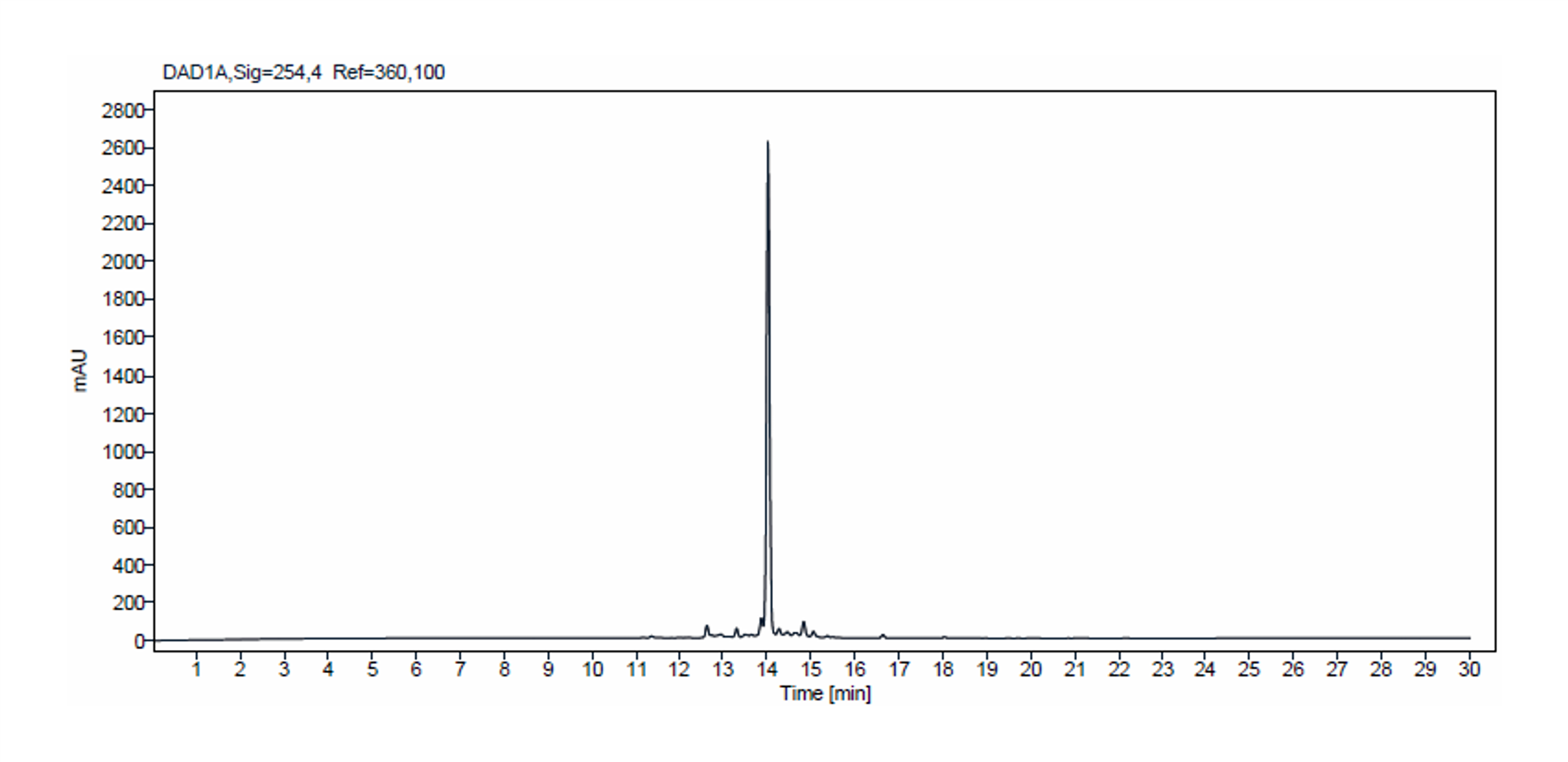

Supplement: S3 Fig — (TIF) [file pone.0317489.s003.tif]

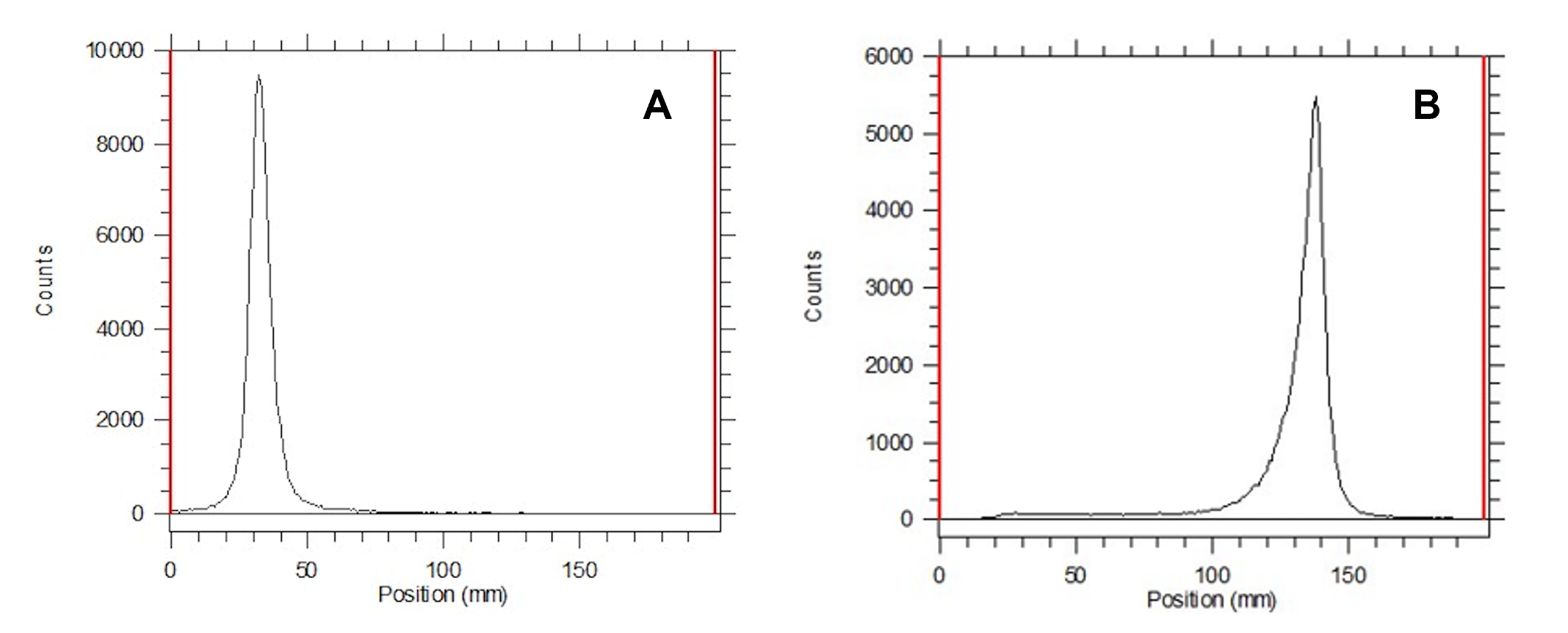

Supplement: S4 Fig — (a) Free 68Ga (b) 68Ga-MI-0202C1. (TIF) [file pone.0317489.s004.tif]

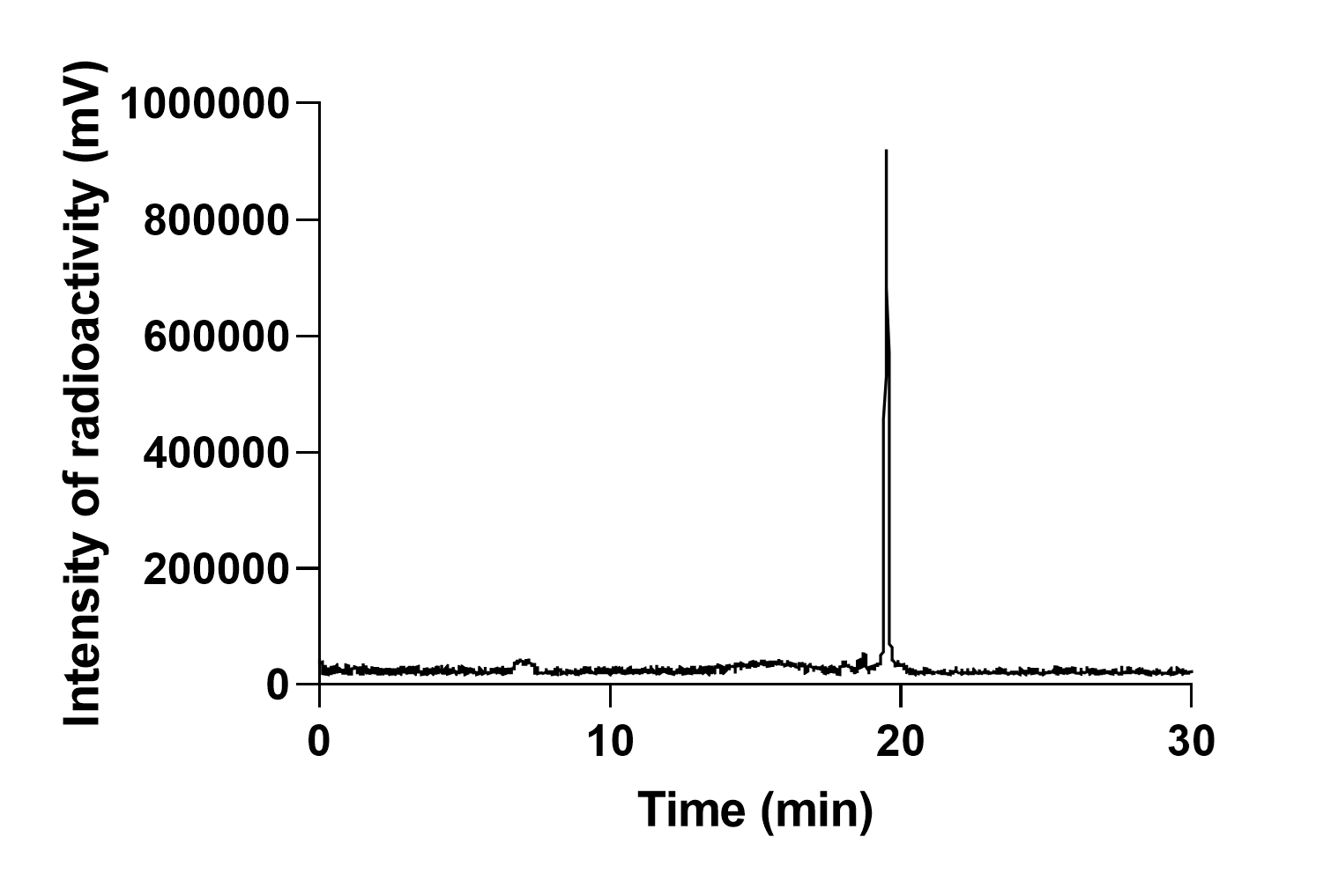

Supplement: S5 Fig — (TIF) [file pone.0317489.s005.tif]

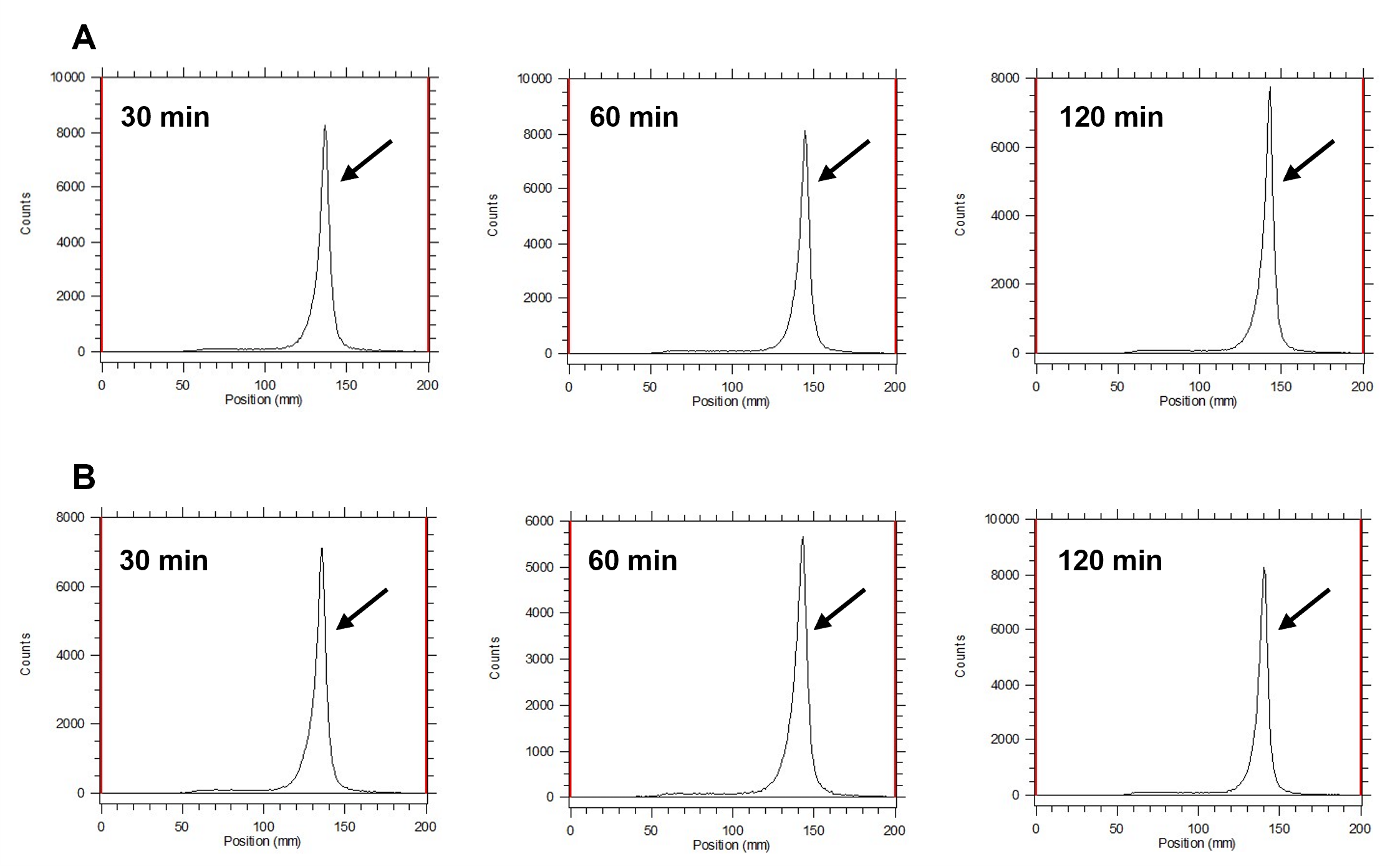

Supplement: S6 Fig — (Arrow: 68Ga-MI-0202C1). (TIF) [file pone.0317489.s006.tif]
