# Supplementary material for: 68Ga-labeled fluorinated benzamide derivatives for positron emission tomography imaging of melanoma
Source: PLoS One. 2025 Feb 28;20(2):e0317489. doi: 10.1371/journal.pone.0317489 (PMC11870364; doi:10.1371/journal.pone.0317489)
Supplement: S1 Table — (DOCX) [file pone.0317489.s007.docx]

**S1 Table.** ***Ex-vivo* biodistribution studies of ^68^Ga-MI-0202C1 in B16F10 tumor-bearing mice at 60 min post-injection**

|  | 60 min |
| --- | --- |
| Blood | 0.38 ± 0.07 |
| Heart | 0.22 ± 0.02 |
| Lung | 0.54 ± 0.17 |
| Liver | 0.94 ± 0.10 |
| Spleen | 0.27 ± 0.06 |
| Stomach | 0.14 ± 0.14 |
| Intestine | 7.61 ± 1.07 |
| Kidney | 1.78 ± 0.44 |
| Pancreas | 0.17 ± 0.07 |
| Normal muscle | 0.13 ± 0.02 |
| Bone | 0.29 ± 0.01 |
| Brain | 0.10 ± 0.02 |
| Skin | 0.32 ± 0.06 |
| Tumor | 0.63 ± 0.10 |
| Tumor-to-blood | 1.69 ± 0.36 |
| Tumor-to-lung | 1.21 ± 0.17 |
| Tumor-to-bone | 2.16 ± 0.32 |
| Tumor-to-brain | 6.47 ± 0.26 |
| Tumor-to-skin | 1.98 ± 0.20 |
